# VitroJet: new features and case studies

**DOI:** 10.1107/S2059798324001852

**Published:** 2024-03-15

**Authors:** Rene J. M. Henderikx, Daniel Mann, Aušra Domanska, Jing Dong, Saba Shahzad, Behnam Lak, Aikaterini Filopoulou, Damian Ludig, Martin Grininger, Jeffrey Momoh, Elina Laanto, Hanna M. Oksanen, Kyrylo Bisikalo, Pamela A. Williams, Sarah J. Butcher, Peter J. Peters, Bart W. A. M. M. Beulen

**Affiliations:** a CryoSol-World, Weert, The Netherlands; b Maastricht Multimodal Molecular Imaging Institute (M4i), Division of Nanoscopy, Maastricht University, Maastricht, The Netherlands; c Ernst Ruska-Centre for Microscopy and Spectroscopy with Electrons (ER-C-3): Structural Biology, Forschungszentrum Jülich, Jülich, Germany; d Institute of Biological Information Processing (IBI-6): Structural Cell Biology, Forschungszentrum Jülich, Jülich, Germany; e Molecular and Integrative Bioscience Research Programme, Faculty of Biological and Environmental Sciences, University of Helsinki, 00014 Helsinki, Finland; f Helsinki Life Science Institute–Institute of Biotechnology, University of Helsinki, 00014 Helsinki, Finland; g Astex Pharmaceuticals, 436 Cambridge Science Park, Milton Road, Cambridge CB4 0QA, United Kingdom; h Institute of Organic Chemistry and Chemical Biology, Goethe University Frankfurt, Frankfurt am Main, Germany; i Department of Nanomedicine and Theranostics, Institute for Experimental Molecular Imaging, RWTH Aachen University, Aachen, Germany; j Department of Biological and Environmental Science, Nanoscience Center, University of Jyväskylä, 40014 Jyväskylä, Finland; University of Leeds, United Kingdom

**Keywords:** cryo-EM, VitroJet, ice thickness, pin printing, jet vitrification

## Abstract

Single-particle cryo-electron microscopy has become a widely adopted method in structural biology due to many recent technological advances in microscopes, detectors and image processing. Before being able to inspect a biological sample in an electron microscope, it needs to be deposited in a thin layer on a grid and rapidly frozen. The VitroJet was designed with this aim, as well as avoiding the delicate manual handling and transfer steps that occur during the conventional grid-preparation process. Since its creation, numerous technical developments have resulted in a device that is now widely utilized in multiple laboratories worldwide. It features plasma treatment, low-volume sample deposition through pin printing, optical ice-thickness measurement and cryofixation of pre-clipped Autogrids through jet vitrification. This paper presents recent technical improvements to the VitroJet and the benefits that it brings to the cryo-EM workflow. A wide variety of applications are shown: membrane proteins, nucleosomes, fatty-acid synthase, Tobacco mosaic virus, lipid nanoparticles, tick-borne encephalitis viruses and bacteriophages. These case studies illustrate the advancement of the VitroJet into an instrument that enables accurate control and reproducibility, demonstrating its suitability for time-efficient cryo-EM structure determination.

## Abstract 2

This paper presents recent technical improvements to the VitroJet and the benefits that it brings to the cryo-EM workflow, as well as a wide variety of case studies. This illustrates the advancement of the VitroJet into an instrument that enables accurate control and reproducibility, demonstrating its suitability for time-efficient cryo-EM structure determination.

## 1. Introduction

As a consequence of major improvements in methods and technology, single-particle cryo-electron microscopy (cryo-EM) has become a mainstream method in structural biology (Henderson & Hasnain, 2023 ▸). It allows the generation of experimental maps of proteins at sufficient resolution to build atomic models, while eliminating the need for protein crystallization (Callaway, 2015 ▸). This has fueled an exponential increase in the number of resolved structures (Renaud *et al.*, 2018 ▸; Callaway, 2020 ▸). Cryo-EM has proved to be applicable to a wide range of sample classes from viruses to filaments, and from small membrane proteins to large protein complexes (Subramaniam *et al.*, 2016 ▸). Its versatility, combined with the capability to resolve structures that exhibit conformational heterogeneity, has led to an increased adoption of cryo-EM in structural biology laboratories (Eisenstein, 2018 ▸; Vinothkumar & Henderson, 2016 ▸). This growth has led to an increasing demand for highly qualified and skilled specialists who are essential to operate these facilities. Simultaneously, there is a need to enhance the robustness of grid preparation, making it more accessible for novice practitioners with limited experience (Mills, 2021 ▸; Walsh *et al.*, 2022 ▸).

Currently, one of the obstacles to obtaining more cryo-EM structures is the limited level of automation and throughput (Zhu *et al.*, 2023 ▸). In X-ray crystallography, synchrotron beamlines are able to screen and collect data from 20 crystals in one hour (Weber *et al.*, 2019 ▸). Many cryo-EM users initially screen the quality of samples one by one in a single grid-loading electron microscope. In larger facilities using a high-end screening microscope, a full cassette of 12 clipped grids can be loaded into the microscope in one go, but screening and acquiring data from these samples can still be a hands-on process due to grid-to-grid variations. Efforts to increase microscopy throughput focus on automated screening for sample integrity and live processing (Cheng *et al.*, 2023 ▸; Punjani, 2020 ▸). Surprisingly, however, grid preparation still often involves several intricate manual handling and transfer steps, including separate devices for glow discharging, vitrification and clipping (Passmore & Russo, 2016 ▸). Damaged grids due to manual handling are frequently excluded only after low-magnification cryo-EM inspection, emphasizing the need for improved monitoring in early stages of sample preparation (Koh *et al.*, 2022 ▸). Ideally, each stage of the cryo-EM workflow from biochemistry to structure determination would be carried out using an easy-to-use instrument with a quality-control mechanism (Nogales, 2016 ▸). Recent innovations in grid preparation include enhanced automation and optical ice-thickness determination, with the aim of selectively loading grids suitable for high-resolution structure determination (Henderikx *et al.*, 2023 ▸; Hohle *et al.*, 2022 ▸; Last *et al.*, 2023 ▸).

The quantity and quality of data sets that can be generated with the current infrastructure over a given period of time is heavily dependent on the composition of the biological samples, the characteristics of the grid and the method of sample deposition and vitrification (Weissenberger *et al.*, 2021 ▸; Drulyte *et al.*, 2018 ▸; Carragher *et al.*, 2019 ▸). Although isolated biological samples are retained in a near-native environment, some samples embedded in vitreous ice still encounter denaturation or adopt preferred orientations when observed via the electron microscope (Shen, 2018 ▸). To reduce degradation, the construct and buffer conditions can be optimized or additives can be added, often aiming to surround and protect the samples from the air–water interface (Sgro & Costa, 2018 ▸; Autzen *et al.*, 2019 ▸). Additionally, sample carriers play an important role, as some particles tend to adhere to the support material. Several developments of grid materials with different surface properties aim to improve particle behavior on the sample carrier (Palovcak *et al.*, 2018 ▸; Huang *et al.*, 2020 ▸; D’Imprima *et al.*, 2019 ▸). Finally, the sample and carrier interact during grid preparation, where the sample is deposited and vitrified. In conventional grid preparation, the biological sample of interest is pipetted onto a grid, followed by blotting with filter paper to remove excess volume, before rapidly plunging the grid into ethane. Although several studies have confirmed ice thickness to be a key parameter in the efficiency and quality of cryo-EM data acquisition, the blotting process lacks the necessary consistency and reproducibility (Kim *et al.*, 2018 ▸; Neselu *et al.*, 2023 ▸; Armstrong *et al.*, 2020 ▸). Therefore, many techniques that provide an alternative to blotting and plunging focus on enhanced control over sample deposition (Ravelli *et al.*, 2020 ▸; Jain *et al.*, 2012 ▸; Arnold *et al.*, 2017 ▸; Koning *et al.*, 2022 ▸). Despite these developments, many conditions usually need to be screened using the microscope before samples of satisfactory quality are obtained (Kampjut *et al.*, 2021 ▸; Xu & Dang, 2022 ▸).

In this contribution, we demonstrate the latest developments in the VitroJet, an instrument aiming to address the abovementioned topics by providing greater ease of use and control over the grid-preparation process. The technical advances from the prototype that we described earlier (Ravelli *et al.*, 2020 ▸) to the current state-of-the-art version are illustrated. These include upgrades of the integrated plasma treatment, sample deposition through pin printing, optical ice-thickness measurement and cryofixation through jet vitrification. Additionally, an overview of the updated operating procedure provides insights into how the VitroJet can be utilized in the cryo-EM workflow. Finally, various case studies including different sample types are presented to demonstrate a range of applications.

## 2. Materials and methods

### 2.1. Membrane protein

The purified membrane protein was mixed with a ligand (10 m*M* stock in 100% DMSO) to form the final protein–ligand complex (final compound concentration of 0.4 m*M* with 4% DMSO). The complex was incubated on ice for 30 min before centrifugation at 13 000*g* at 4°C for 10 min to remove any precipitation. Prior to vitrification, UltraAuFoil R1.2/1.3 300 mesh gold grids were clipped at room temperature and then plasma-cleaned in the VitroJet for 60 s. The protein–ligand sample was pin-printed on the gold film in a circle spiral pattern at a speed of 2 mm s^−1^ with a spacing of 70 µm and a standoff distance of 10 µm. After printing, the Autogrids were jet vitrified and the samples were stored under liquid nitrogen until used for data collection.

Two data sets were collected on a Thermo Fisher Scientific Krios G4, which was equipped with a Falcon 4i detector. The microscope was operated in counting mode at a nominal magnification of 120 000×, with a pixel size of 0.64 Å. Initially, micrographs from two data sets were processed separately using our in-house *WebCryo* pipeline until 2D classifications were completed (Saur *et al.*, 2020 ▸). Selected particles obtained from the two 2D classifications were then combined and exported to *cryoSPARC* for an additional round of 2D classification, followed by a four-class *ab initio* reconstruction (Punjani *et al.*, 2017 ▸). The highest-quality particle class resulting from the *ab initio* reconstruction was then subjected to heterogeneous refinement and non-uniform refinement, ultimately yielding a final density with a resolution of 2.7 Å.

### 2.2. Nucleosome

Nucleosome samples at approximately 2 mg ml^−1^ in a buffer consisting of 20 m*M* HEPES pH 7.5, 1 m*M* EDTA, 0.5 m*M* TCEP were frozen on Quantifoil 200 mesh R2/1 grids. Optimal grids were produced with the following settings: 30 s plasma-cleaning, grid and pin dew-point offsets of −0.1°C and 0.6°C, respectively, a pin standoff distance of 10 µm and a square spiral deposition pattern composed of six spirals at a distance of 0.1 mm and a velocity of 1.0–1.5 mm s^−1^.

Data were collected on a Titan Krios G3 fitted with a Gatan K3 camera with post-energy filter using the automated acquisition software *SerialEM*. Images were processed in *cryoSPARC*, resulting in a reconstruction that extended to approximately 3.0 Å, with particles exhibiting slightly less orientation bias when compared with grids produced on a Vitrobot.

### 2.3. Fatty-acid synthase

Fatty-acid synthase was purified to a concentration of 2.1 mg ml^−1^ in 100 m*M* potassium phosphate pH 6.5 buffer as described previously (Joppe *et al.*, 2020 ▸; Gajewski *et al.*, 2017 ▸; Chakravarty *et al.*, 2004 ▸). Deposition onto Quantifoil R2/1 Cu 200 grids did not yield enough particles for structure determination due to absorption of the protein by the carbon foil. Therefore, pre-clipped UltrAuFoil R1.2/1.3 Au 300 grids were externally glow-discharged for 60 s in a Pelco EasiGlow device for grid cleaning prior to a second round of plasma-cleaning for 60 s in the VitroJet. VitroJet pins were cleaned with detergent and 70% ethanol in an ultrasonic bath (5 min each), followed by 1 min drying under a nitrogen stream. Freshly purified fatty-acid synthase was pin-printed at a climate chamber temperature of 4°C with a 70 µm spaced spiral, 15 µm standoff and a velocity of 5 mm s^−1^.

A total of 3696 micrographs representing the complete deposition area were recorded using a 200 kV Talos Arctica microscope equipped with a Gatan K3 BioQuantum energy-filtered detector with a super-resolution pixel size of 0.408 Å using one shot per hole and a total fluence of 48 e^−^ Å^−1^. Micrographs were processed in* cryoSPARC Live* version 4.2.1 (Punjani *et al.*, 2017 ▸, 2020 ▸). After patch motion correction (binning to physical pixel size), patch CTF correction, blob picking with a diameter of 260–320 Å and 2D classification of 10 000 particles into 20 classes to generate 2D templates, template matching was performed with default parameters. The best classes from streaming 2D classification were selected for *ab initio* 3D model generation (45 979 particles, no symmetry) and refined with *D*3 symmetry using non-uniform refinement optimizing per-particle defocus and group CTF parameters (tilt, trefoil, spherical aberration and tetrafoil) and minimizing on a per-particle scale to a final resolution of FSC(0.143) = 3.3 Å. Local resolution estimation and local filtering was performed in *cryoSPARC* using default parameters. Heterogeneous refinement into three classes with *C*1 symmetry was performed and the resulting volumes were visually inspected for air–water interface damage. Images were created in *ChimeraX* with PDB entry 6ta1 docked. The reconstructed cryo-EM map was uploaded to the Electron Microscopy Data Bank (EMDB) as entry EMD-19477 and the raw micrographs were uploaded to EMPIAR with accession No. EMPIAR-11873.

### 2.4. Lipid nanoparticles

Lipid nanoparticles (LNPs) are designed to encapsulate siRNA, which aims to disrupt the expression of NEMO/IKK-γ mRNA. LNP samples were prepared on Quantifoil 1.2/1.3 Cu 200 grids with a 2 nm continuous carbon support film. The grids were processed in the VitroJet using 30 s plasma-cleaning and were deposited at velocities ranging from 2 to 5 mm^−1^ using a standoff distance of 10–15 µm and a spiral spacing of 70–150 µm.

Imaging was carried out on a 200 kV Talos Arctica equipped with a K3 BioQuantum energy filter at a pixel size of 2.79 Å, with a nominal electron dose of approximately 80 e Å^−2^, within a defocus range of −5 to −20 µm.

### 2.5. Tobacco mosaic virus (TMV)

TMV was prepared at a concentration of 20 mg ml^−1^ in distilled water as described previously (Fromm *et al.*, 2015 ▸), snap-frozen in liquid nitrogen and stored at −80°C. Pre-clipped Quantifoil R2/1 grids were externally glow-discharged for 60 s in a Pelco EasiGlow device for grid cleaning prior to a second round of plasma-cleaning for 60 s in the VitroJet. VitroJet pins were cleaned with detergent and 70% ethanol in an ultrasonicator followed by 1 min drying under a nitrogen stream (5 min each). TMV was pin-printed at a 4°C chamber temperature with a 70 µm spaced spiral and 15 µm standoff with a velocity of 5 mm s^−1^.

iDPC–STEM micrographs of cryo-samples were recorded as described previously on a 300 kV Titan Krios G4 equipped with HAADF STEM and Panther STEM detectors (Lazić *et al.*, 2022 ▸). Briefly, after probe alignment and calibrating CSA = 2.0 mrad with a gold cross-grating grid, a low-resolution atlas of the TMV cryo-specimen was recorded via the HAADF detector in the *MAPS* software for grid navigation. Focusing and stigmation were performed via the ronchigram on the FluScreen on carbon. 103 iDPC–STEM images were recorded with a pixel size of 1.3 Å on a 4k × 4k detector with a total fluence of 37 e^−^ Å^−1^ in the *Velox* software. Micrographs were Gaussian high-pass filtered with a full width at half maximum (FWHM) of 251 Å and imported as negative-stain images (no contrast flipping during particle extraction) and constant CTF into *cryoSPARC* version 4.2.1 (Punjani *et al.*, 2017 ▸). Particles were picked directly from the imported micrographs using the internal filament tracer, with a filament width of 250 Å and a separation distance of 83 Å. Best classes from 2D classification with default parameters were selected and subjected to helical refinement (13 644 particles) with a helical twist estimation of 22.03° and a helical rise estimation of 1.4 Å, converging to final helical parameters of twist = 22.03° and rise = 1.474 Å to a resolution of FSC(0.143) = 5.6 Å. Helical rise was used to recalibrate the pixel size of 1.3 Å to the corrected pixel size of 1.25 Å, resulting in a slight increase in the resolution of FSC(0.143) = 5.4 Å. The reconstructed cryo-EM map was uploaded to the EMDB as entry EMD-19489 and the raw micrographs were uploaded to EMPIAR with accession No. EMPIAR-11874.

### 2.6. Viruses

Purified tick-borne encephalitis virus was deposited onto pre-clipped Quantifoil 1.2/1.3 grids with a 2 nm continuous carbon layer using 150 µm diameter pins. Bacteriophage FJy-3 was purified from its host, *Flavobacterium* sp. B169, in 20 m*M* potassium phosphate pH 7.2. The sample was deposited onto pre-clipped Quantifoil holey carbon R1.2/1.3 Cu 300 mesh grids in the VitroJet. Prior to sample deposition, the grids were plasma-cleaned in the VitroJet for 75 s. For pin printing, a circular spiral pattern was used with a speed of 1 mm s^−1^, a spacing of 120 µm and a standoff distance of 10 µm.

Cryo-EM grid screening and data collection were performed at the cryo-EM facility in Helsinki, Finland using a Thermo Fisher Scientific Talos Arctica operating at 200 kV and equipped with a Falcon 3 direct electron detector operating in linear mode. Images were collected at 57 000× magnification with an image size of 4096 × 4096 at a sampling rate of 0.26 nm per pixel.

## 3. Results

### 3.1. Technical improvements

#### 3.1.1. Integration

**Figure 1:**
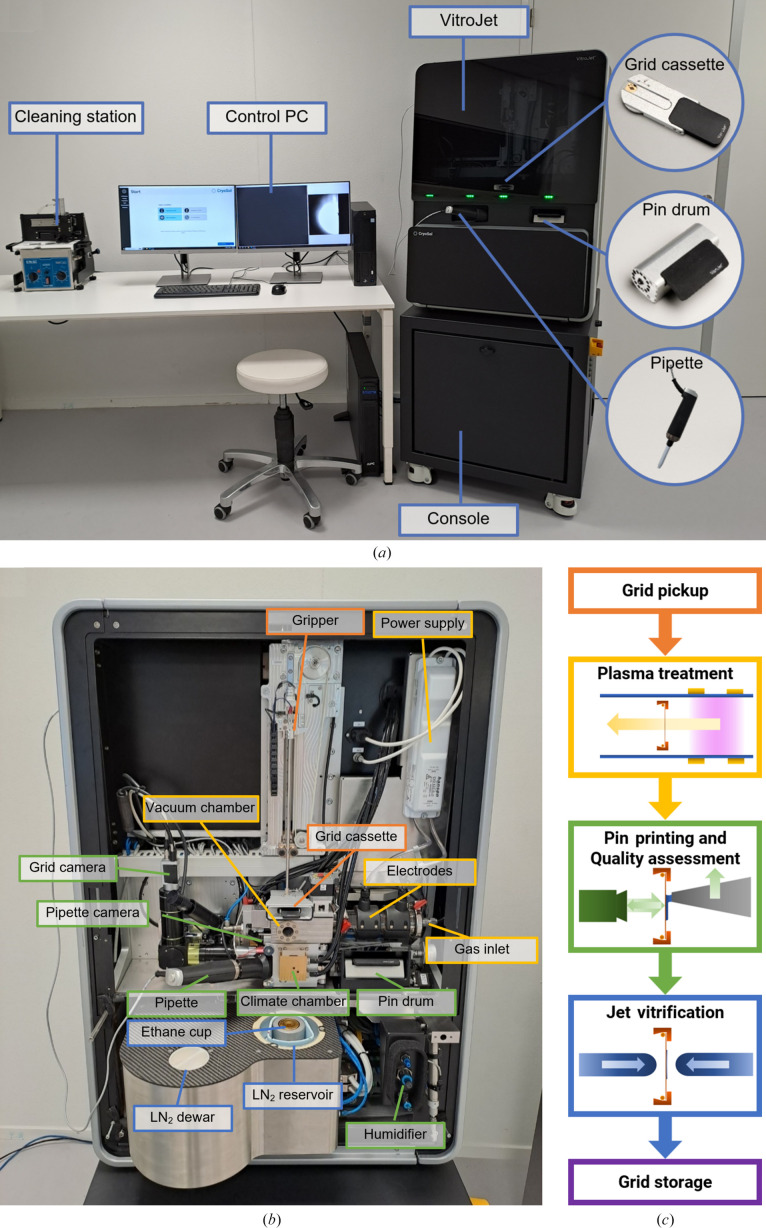
Overview of the VitroJet setup including accessories, components inside the instrument and process steps. (*a*) Overview of the VitroJet, which can be divided into a console and the main instrument. The control PC is used to operate the system and the reusable deposition pins can be cleaned ultrasonically in a separate cleaning station. (*b*) The VitroJet without front covers and (*c*) a schematic representation of the steps that are performed to process one Autogrid. Firstly, the gripper picks up one of the Autogrids from the grid cassette for transportation through the grid-preparation process. Secondly, the Autogrid is placed in the vacuum chamber, where the pressure is regulated using the gas inlet. The power supply applies a potential difference to the electrodes to generate a plasma. The plasma is generated remotely from the grid, and the flow towards the grid provides gentle cleaning to make it hydrophilic. Thirdly, the Autogrid is brought down to the temperature-controlled climate chamber where the humidifier provides an environment for dew-point regulation. A pin from the pin drum picks up sample from the pipette and scribes it onto the grid by pin printing. The pin is maintained at a distance from the Autogrid and moved laterally with respect to the grid to deposit a thin layer. These processes are monitored using the pipette and grid cameras. Fourthly, the Autogrid is jet vitrified in the ethane cup by two jets that target the center of the Autogrid to cool the sample. Finally, the Autogrid is automatically stored in the grid boxes located in the liquid-nitrogen (LN_2_) reservoir, where the level of liquid nitrogen is maintained through the liquid-nitrogen dewar.

The VitroJet consists of the instrument itself, a control PC and a cleaning station (Fig. 1 ▸ *a*). It is divided into two enclosed sections for protection against the environment and safe user operation. The console, the stable base, holds power sources, an uninterruptible power supply and a vacuum pump. The main instrument, located above, contains functional modules to process the grids (Fig. 1 ▸ *b*). The VitroJet is compatible with pre-clipped Autogrids, which are assemblies of an EM grid mounted in a sturdy ring to enable automated handling. The EM grid itself is a sample carrier that is commonly used in electron microscopy, consisting of a metal mesh and a perforated foil. Different types of materials and geometries for the mesh and foil hole pattern are available. These grids are clipped into a sturdy ring by means of a c-clip, after which the assembly is called an Autogrid. When using the VitroJet, grids are pre-clipped into an Autogrid before insertion into a dedicated grid cassette, providing a safe location in the system for up to 12 pieces. From this cassette, the gripper picks up Autogrids one by one, transports them through the entire process and stores them in grid boxes. For each grid-preparation cycle, one grid is processed as explained below (Fig. 1 ▸ *c*).

#### 3.1.2. Plasma treatment

After an Autogrid has been picked up from the grid cassette, it is introduced into the plasma module. The Autogrid is plasma-treated to remove surface contamination and to make it hydrophilic. For each preparation cycle, the Autogrid is positioned in the vacuum chamber and the plasma is ignited for a duration that is predefined by the user. Once this period has elapsed, the chamber is vented and the Autogrid is transported to the climate chamber by the gripper.

The plasma module consists of the plasma source and a vacuum chamber in which the Autogrid is positioned. The plasma source is composed of a glass tube enclosed by high-voltage electrodes, a high-voltage power supply and a mass-flow controller. As an improvement over the prototype, stainless-steel and glass materials were selected for the vacuum chamber and plasma source to prevent sputtering of materials (Fischione *et al.*, 1997 ▸). In the glass tube, ions will accelerate back and forth between the electrodes due to the alternating potential difference. Depending on the voltage, the ions accelerate until either the polarity of the electrodes is switched or an ion bumps into another molecule. In this setup, the voltage and frequency are fixed at 2 kV and 20 kHz, respectively. The Autogrid is positioned at a distance from the plasma to prevent ion bombardment of the grid. In this configuration primarily radicals will reach the grid, resulting in a gentler treatment. The pressure in the plasma cleaner influences the number of molecules in the vacuum chamber, and thus the potential number of active species and the time between interactions. In the VitroJet, the pressure in the vacuum chamber is actively controlled by regulating the gas supply using a mass-flow controller in a proportional–integral–derivative (PID) feedback loop. A gas cylinder can be connected to the system to further optimize plasma treatment and to establish compatibility with different grid types. Commonly, a cylinder with mixture of dry nitrogen and oxygen is provided to eliminate any fluctuations in plasma performance occurring from possible variations in the humidity of the environmental air. Alternatively, mixtures of argon and oxygen in different ratios can be connected, where lower percentages of oxygen can provide a gentler cleaning process. This can be helpful when working with delicate sample carriers such as continuous graphene films. Finally, the plasma-cleaning process can be bypassed, for example to maintain the properties of functionalized films.

#### 3.1.3. Pin printing

After plasma-cleaning, the gripper positions the Autogrid in the deposition module. Here, the climate chamber is designed to provide well defined conditions for performing sample deposition that are not impacted by ambient conditions. After deposition, the Autogrid is rapidly transferred into the cryo-module for vitrification.

In the prototype, the temperature during sample deposition was not actively regulated and sample deposition occurred at room temperature. Additionally, entry ports for components such as the pin, gripper and pipette had limited sealing, leading to potential disturbance of the deposition environment. The current climate chamber has improved sealing from the environment and allows sample deposition both at 4 and 20°C, since many single-particle samples are most stable at 4°C. To avoid evaporation from or condensation onto the grid, the temperatures and/or humidities of different components of the climate chamber are actively controlled. The climate chamber is continuously supplied with saturated nitrogen gas to ensure reproducible conditions and potentially reduce oxidation. To achieve dew-point temperature, the nitrogen gas is humidified upon entering the VitroJet and is subsequently cooled in a condenser to the desired deposition temperature. The nitrogen gas exiting the condenser is at the dew point, which means that it is at a relative humidity of 100%. The walls of the climate chamber are regulated to a temperature 0.2°C above the dew point, thus avoiding condensation on the walls and preserving the dew-point temperature. To mitigate evaporation of the deposited layer, the Autogrid and pin are pressed against an element which is temperature-controlled. The temperature of the pin and Autogrid can be controlled with respect to the dew-point temperature to balance between condensation and evaporation. The exact dew point is dependent on sample constituents such as the salt concentration, although it is comparable for most aqueous sample solutions. A motorized pipette is inserted into the climate chamber to introduce the sample, which can be retained or exchanged for subsequent grids. The end of the pipette tip is positioned in the wall of the climate chamber wall to maintain the sample close to the set deposition temperature.

The principles behind pin printing remain the same as in the prototype (Ravelli *et al.*, 2020 ▸). A solid pin dips into the pipette tip containing 0.5 µl sample stock volume, and upon retraction it draws out a droplet of approximately 0.5 nl that remains on the front face of the pin. The pin then approaches the grid to a predefined standoff distance, where the sample forms a liquid bridge between the pin and the grid. Moving the pin laterally with respect to the grid results in the deposition of a thin layer, where thickness, among other properties, can be modulated by the moving velocity and the standoff distance between the pin and grid. In the current VitroJet version, the pin diameter is increased to 150 µm, several writing patterns are available and multiple application is possible to increase the flexibility and coverage. Motors with enhanced positioning accuracy were selected to maintain the standoff distance between the pin and grid and to have a smooth writing motion. A circular spiral can be used to cover an area with a diameter of 800 µm while maintaining a constant writing velocity.

The pins are reusable and need cleaning after each deposition to prevent cross-contamination between subsequent samples. In the prototype, the pins needed to be replaced and cleaned manually after each deposition. To allow the preparation of 12 Autogrids in a single grid-preparation session, a pin drum was developed that holds 12 pins. For each cycle, a pin is automatically loaded from the pin drum and introduced into the climate chamber. After regulating the pin tip to the pre-defined temperature, the sample is picked up from the pipette and deposited onto the grid. Subsequently, the pin is retracted back into the pin drum. The VitroJet is provided with a cleaning station that allows the simultaneous cleaning of all pins in the pin drum by sonication in a detergent solution and 70% ethanol and finally drying with nitrogen gas.

#### 3.1.4. Quality assessment

**Figure 2:**
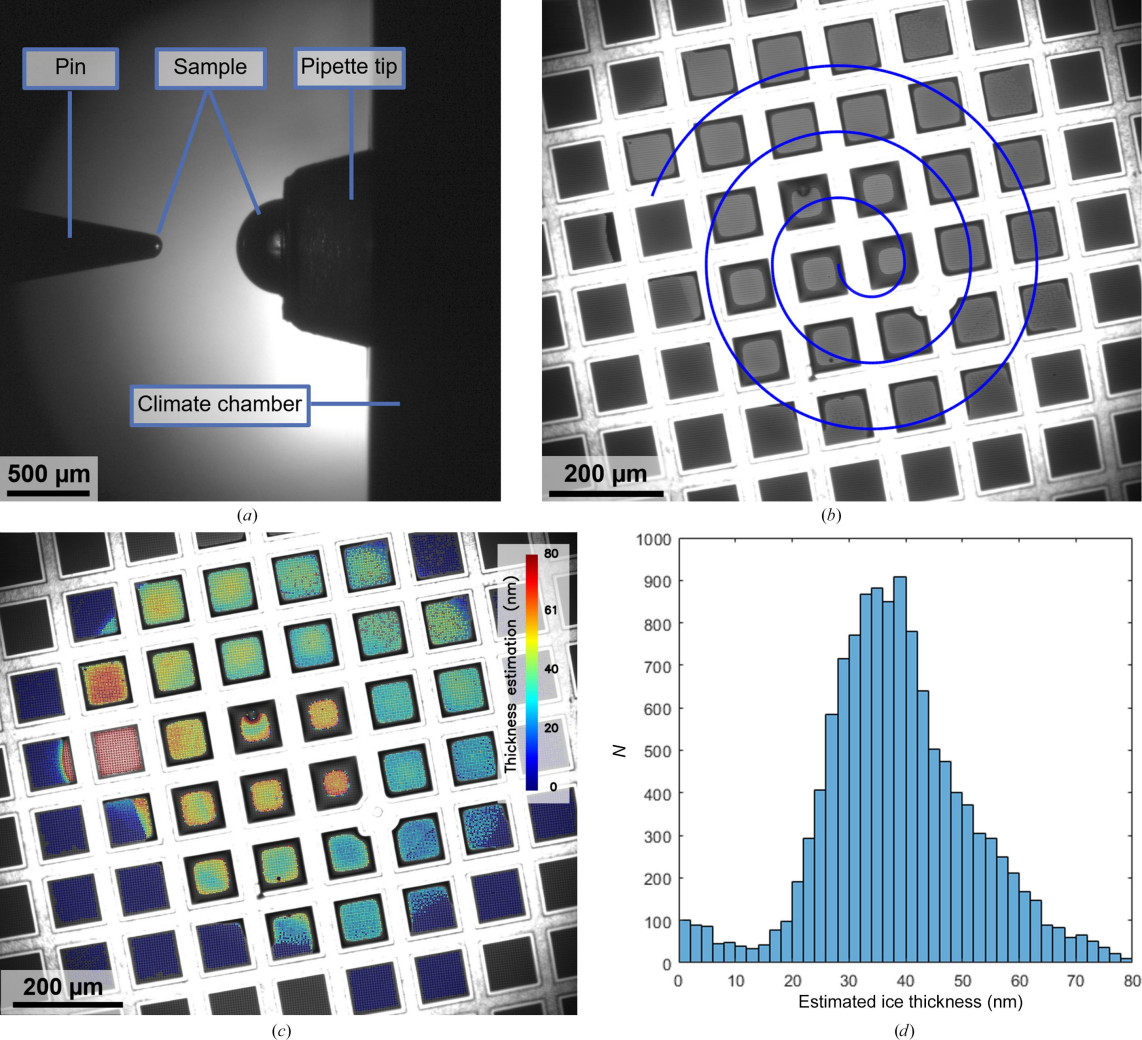
Images from the VitroJet cameras to assess the sample pickup and deposition process and the resulting estimation of ice thickness. (*a*) Image from the pipette camera after droplet pickup. The sample is presented as a half sphere at the tip of the pipette, after which the solid pin dips into the sample. Upon retraction, a sample volume of approximately 0.5 nl is located on the front face of the pin. The pipette tip is situated in the temperature-controlled wall of the climate chamber. (*b*) Image from the grid camera to monitor sample deposition by pin printing. In this example, the pin started in the center of the field of view and spiraled outwards to cover an area with a diameter of 800 µm on a 200 mesh R2/1 grid. The intensity of the deposited layer indicates the thickness of the layer. (*c*) Color overlay of the estimated ice thickness for each individual hole based on the grid-camera images during deposition. (*d*) Histogram of the estimated thicknesses for the holes in the perforated foil, with a total of 12 093 holes detected in the squares where the sample was deposited.

Two cameras are integrated into the deposition module of the VitroJet to monitor the grid-preparation process, which record images of the grid and the pipette. The pipette camera visualizes the sample that is located in the pipette tip and is used to monitor and control the extrusion of the sample droplet before pickup by the pin (Fig. 2 ▸ *a*). The grid camera is used to determine the relative position of the front face of the pin and the foil of the grid, which is required for positioning them at the desired standoff distance for pin printing (Fig. 2 ▸ *b*). Additionally, the cameras can be utilized for dew-point offset calibration to adjust the temperature setpoints of the pin and grid during deposition.

Obtaining images of the deposition process before loading grids into the electron microscope has already proved its value in the prototype. In the current setup, we further improved the employed optical components to obtain deposition images with an increased resolution frame rate and field of view. A monochromatic camera was implemented to enhance sensitivity.

The sample pickup by the pin is visualized by the pipette camera (Supplementary Movie S1). From these images, the sample volume on the pin can be determined using the pin diameter and droplet height. In this case, the volume of the spherical cap on the pin is approximately 0.5 nl. Subsequently, it was deposited after 60 s of plasma-cleaning in a circular spiral with a writing velocity of 1 mm s^−1^, a standoff distance of 10 µm and a spiral spacing of 120 µm. The deposition process is monitored by the grid camera (Supplementary Movie S2), which records 3.7 s with these parameters. During deposition, particles risk interacting with the air–water interface, since the diffusion time in layers suitable for cryo-EM is estimated to be <1 ms (Taylor & Glaeser, 2008 ▸; Naydenova & Russo, 2017 ▸). The chosen protocol parameters result in a coverage of 30 squares on a 200 mesh grid, which would be equivalent to approximately 70 squares on a 300 mesh grid.

The grid is illuminated by white light that reflects from the grid and sample before being captured by the camera. On grids that are plasma-cleaned and hydrophilic, sample accumulates in the corners between the grid mesh and perforated foil and appears as a dark ring around the squares. The center of each of the squares shows a uniform color, where depending on thickness the reflected light from both interfaces can constructively or destructively interfere. Based on these optical camera images, we developed an algorithm to estimate the ice thickness during grid preparation. The algorithm uses the normalized intensity difference for each individual hole before and after deposition to calculate the thickness from thin-film interferometry (Henderikx *et al.*, 2023 ▸). The output can be displayed as a color overlay on the deposition to assess the spatial distribution of thicknesses (Fig. 2 ▸ *c*) or as a histogram to evaluate the number of holes with a certain thickness (Fig. 2 ▸ *d*). Based on the analysis in this example, it is estimated that sample was deposited in 12 093 holes with a diameter of 2 µm and an interspacing of 1 µm.

#### 3.1.5. Jet vitrification

After deposition, the Autogrid is rapidly transported from the climate chamber into the cryo-module to be jet vitrified. The transfer time between deposition and vitrification is minimized to avoid evaporation and is currently 95 ms. Two ethane jets that are temperature controlled to −180°C target the sample in the center of the Autogrid, enabling vitrification of pre-clipped Autogrids. After vitrification, each Autogrid is automatically stored in a grid box.

Although the concept behind jet vitrification remained the same as in the prototype, the automation of the module was improved. An integrated 2 l liquid-nitrogen dewar is used to maintain adequate liquid-nitrogen levels in the reservoir for the storage of processed Autogrids in grid boxes. Gaseous ethane is automatically condensed in the ethane cup at the start of each experiment and refilled after each jet vitrification. The volume of liquid nitrogen allows operation for approximately 90 min to cater for the vitrification and storage of 12 Autogrids without manual interaction, aiming to reduce contamination. The cryo-module is situated in a closed compartment at the bottom of the main instrument to shield the process from the environmental conditions. At the end of the session, the door opens and provides access for the user to retrieve the grid boxes.

### 3.2. Workflow

#### 3.2.1. VitroJet operation

**Figure 3:**
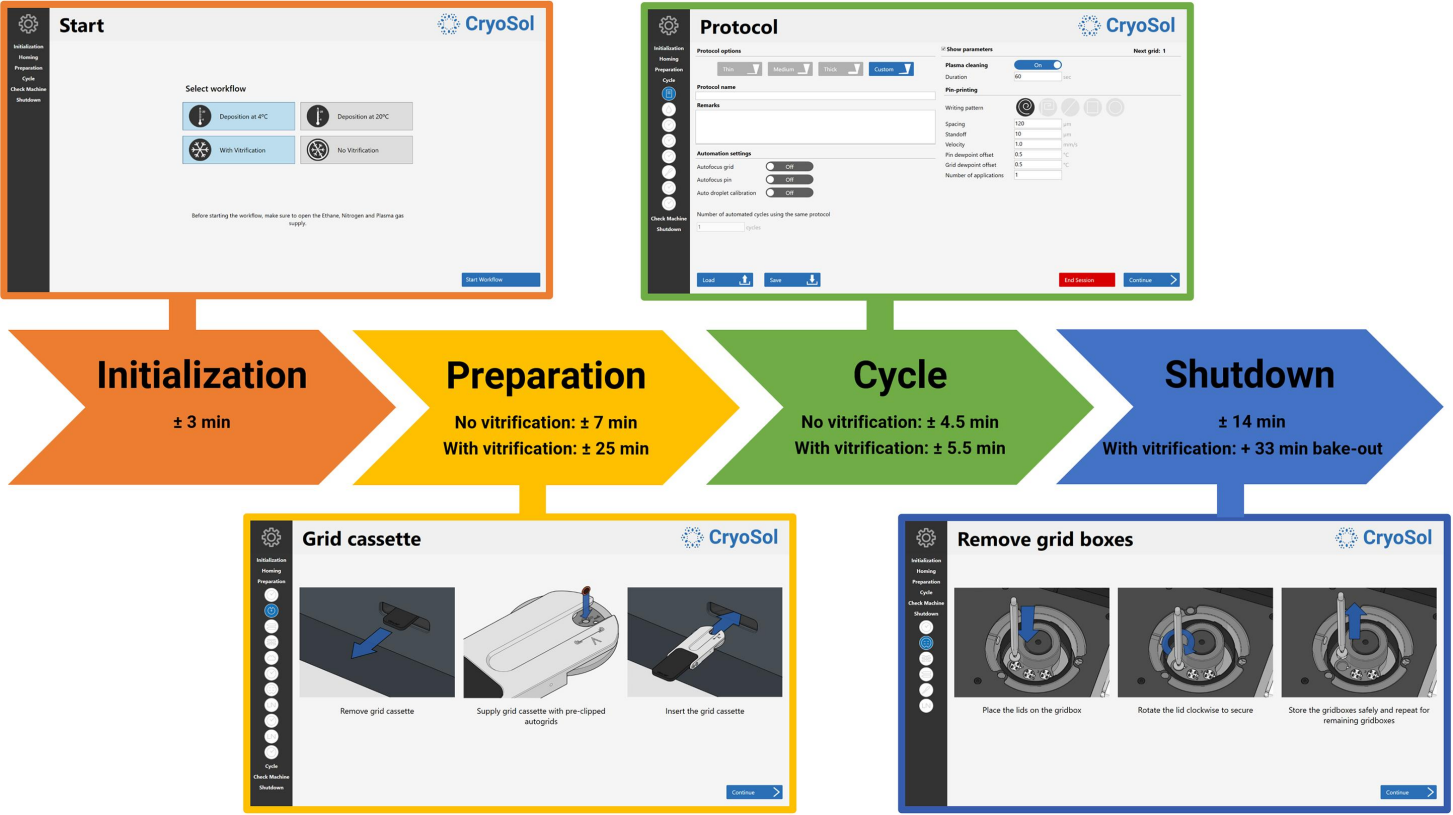
Schematic overview of the grid-preparation workflow in the VitroJet. Using the control PC and the user interface, the operator is guided through the process. Durations for each stage of the workflow are indicative, depending on the selected settings. After selection of the deposition temperature and the decision on whether to perform vitrification, the system starts an initialization procedure. Subsequently, in the preparation phase the user is presented with instructions to prepare the system for processing up to 12 pre-clipped Autogrids. Once the preparation has finished, the protocol settings can be defined for each grid separately, and the instrument is able to automatically process Autogrids one by one. Once sufficient grids have been prepared, the operator is guided through a shutdown procedure to take out the grid boxes and finish the experiment.

The VitroJet prototype still required some manual steps, such as camera focusing, exchanging pins and maintaining cryogen levels. To enhance reproducibility and to limit the amount of operator training that is required, the grid-preparation cycle is now automated. Additionally, the software has a graphical user interface that guides users through each step in the process, which can be roughly divided into four stages (Fig. 3 ▸).

In the first stage of the process, the type of experiment is selected (Fig. 3 ▸, Initialization panel). The deposition temperature can be set to 4 or 20°C, depending on sample preference. Additionally, the user can choose to perform a vitrification or to not cool to cryogenic temperatures. An experiment without vitrification can be used to optimize the settings to achieve a desirable layer thickness based on the feedback from the camera, possibly only using the buffer to save precious sample. In this case, time is saved since the cryo-module remains at room temperature while fine-tuning the deposition settings. According to the experiment-type selection, initialization and homing of the system is started, and the system state and reference positions are automatically checked.

In the preparation stage, the user prepares the system to process up to 12 Autogrids according to instructions in the user interface (Fig. 3 ▸, Preparation panel). Firstly, grids that are pre-clipped in an Autogrid ring are placed in the grid cassette. The grid cassette can be rotated to insert the Autogrids. Next, the pin drum is cleaned in the separate cleaning station by subsequent sonication for 5 min in a detergent solution and 70% ethanol, before drying with nitrogen gas for 2 min. Subsequently, the level of the humidifier is replenished and water accumulated in the condenser is drained. Finally, the grid boxes are inserted into the liquid-nitrogen reservoir and the dewar is filled with liquid nitrogen when performing a vitrification experiment.

Once the preparation has finished, the user defines the settings that will be used to process an Autogrid. The protocol page allows the saving and loading of protocols which include settings for plasma treatment and deposition (Fig. 3 ▸, Cycle panel). Before the grid-processing cycle starts, one can choose whether to load a new sample in the pipette or use the sample that was loaded in a previous cycle. Protocol parameters that can be set in the VitroJet include the following.

(i) Plasma-treatment toggle: switched on for plasma-cleaning in the VitroJet or switched off in the case where special pre-treatment of grids is required, for example carriers with continuous support films and functionalization.

(ii) Plasma-treatment duration (0–300 s): the plasma-cleaning time determines the hydrophilicity of the grid. Longer cleaning durations increase the hydrophilicity of the grid, leading to lower contact angles and increased sample spreading.

(iii) Pin printing number of applications (1–10): the number of depositions on a single grid, which can be used to increase the thickness by applying sample multiple times in the same pattern.

(iv) Figure type (line, square, circle, circular spiral, square spiral): the pattern in which the sample is pin printed onto the grid; used to influence the coverage of the grid.

(v) Spacing (50–200 µm): the distance between the lines of the spiral deposition patterns. A smaller spacing results in overlap between subsequent spirals and redistribution of sample, creating a more uniform thickness.

(vi) Standoff (1–100 µm): the distance that is maintained between the pin and the grid while pin printing, where smaller distances result in thinner layers.

(vii) Velocity (0.1–10 mm s^−1^): the speed with which the pin is moved during deposition. The sample is located between the pin and the grid and follows the movement of the pin. At low velocities, the sample can follow the pin well, leaving only a thin layer on the grid, whereas increasing the velocity leads to a thicker layer.

(viii) Pin/grid dew-point offset (−1 to +1°C): refers to the increase (positive) or decrease (negative) of the temperature with respect to the expected dew point.

Upon starting the cycle, the system automatically processes an Autogrid using the protocol settings and progress can be monitored in the user interface. If the option to reload a sample in the pipette is selected, the user is instructed to do so at the last step before deposition. The quality of sample pickup and deposition can be assessed using the camera. When processing grids with vitrification, a cycle with default settings takes approximately 5.5 min (plasma-treatment duration 60 s, single application in a circular spiral with a spacing of 120 µm, a standoff of 10 µm and a velocity of 1 mm s^−1^). Usually, the velocity and standoff parameters are used to modulate the thickness of sample deposition.

After 12 Autogrids have been processed, or if the user decides to finalize the experiment earlier, the shutdown sequence is initiated. Firstly, the user is prompted to take the grid boxes with vitrified Autogrids from the liquid-nitrogen reservoir and store them safely (Fig. 3 ▸, Shutdown panel). The user then drains any condensed water from the condenser, cleans the pin drum and disposes of the pipette tip. In the case of a vitrification, a bake-out procedure taking approximately 33 min automatically starts, in which the liquid-nitrogen reservoir and ethane cup are heated and dried.

#### 3.2.2. Grid preparation in a cryo-EM workflow

Following grid preparation, the Autogrids are usually screened in the electron microscope to determine whether the samples will be used for data collection. The screening process involves the assessment of a number of criteria. Some of these are dependent on the combination of sample biochemistry, sample carriers and grid preparation, such as particle distribution, concentration, intactness and orientation distribution. Other quality standards can be attributed directly to the grid-preparation process, such as the intactness of the foil, the amount of contamination, the vitreousness of the ice and the ice thickness. With the technical improvements, the VitroJet aims to provide a process that covers the grid-preparation standards, enabling focus to be shifted to biochemical aspects and particle properties, leading to a better understanding of cause and effect of the sample-related criteria.

Regarding sample carriers, the VitroJet is compatible with all grid types that can be pre-clipped into an Autogrid ring. This accommodates grid-type preferences considering sample behavior and cryo-EM imaging. A volume of 0.5 µl is loaded into the pipette tip as a stock, while the pin uses 0.5 nl for deposition. The operator can select whether to load a new sample before starting to process each grid. Therefore, it is possible to only load stock volume in the pipette once and prepare 12 grids with it, or to exchange it for each grid and prepare 12 different samples.

The deposition images can be analyzed to yield an estimation of ice thickness based on optical intensity. This method is demonstrated to determine ice thickness in individual 2 µm holes in the range 0–70 nm with an error below ±20 nm and down to ±10 nm in the range 10–40 nm. This information can be used to optimize protocol parameters without the use of an electron microscope. Additionally, one can use the information to localize regions on EM grids that are most promising, both minimizing the electron microscopy beam time required for screening and saving on computational resources for data processing (Henderikx *et al.*, 2023 ▸).

### 3.3. Case studies

Multiple laboratories have used the VitroJet to test its suitability for their samples and workflow. Several case studies from different users are presented below to show a variety of sample types processed with the VitroJet. For each sample, the motivation to conduct the experiment and resulting outcomes are depicted.

#### 3.3.1. Membrane protein

In the industrial setting, achieving consistently high-quality grids is imperative for solving hundreds of complex structures of specific drug targets. While blotting methods have proven to be successful in obtaining high-resolution structures, there remains ample room for enhancing automation and ensuring consistency in the grid-preparation process. Traditional cryo-EM sample preparation involves multiple manual steps, demanding significant experience and training to produce high-quality grids. To test the applicability of the VitroJet in high-throughput settings, a membrane protein with molecular weight of around 100 kDa and a dimension of 10 nm was processed as a test case.

**Figure 4:**
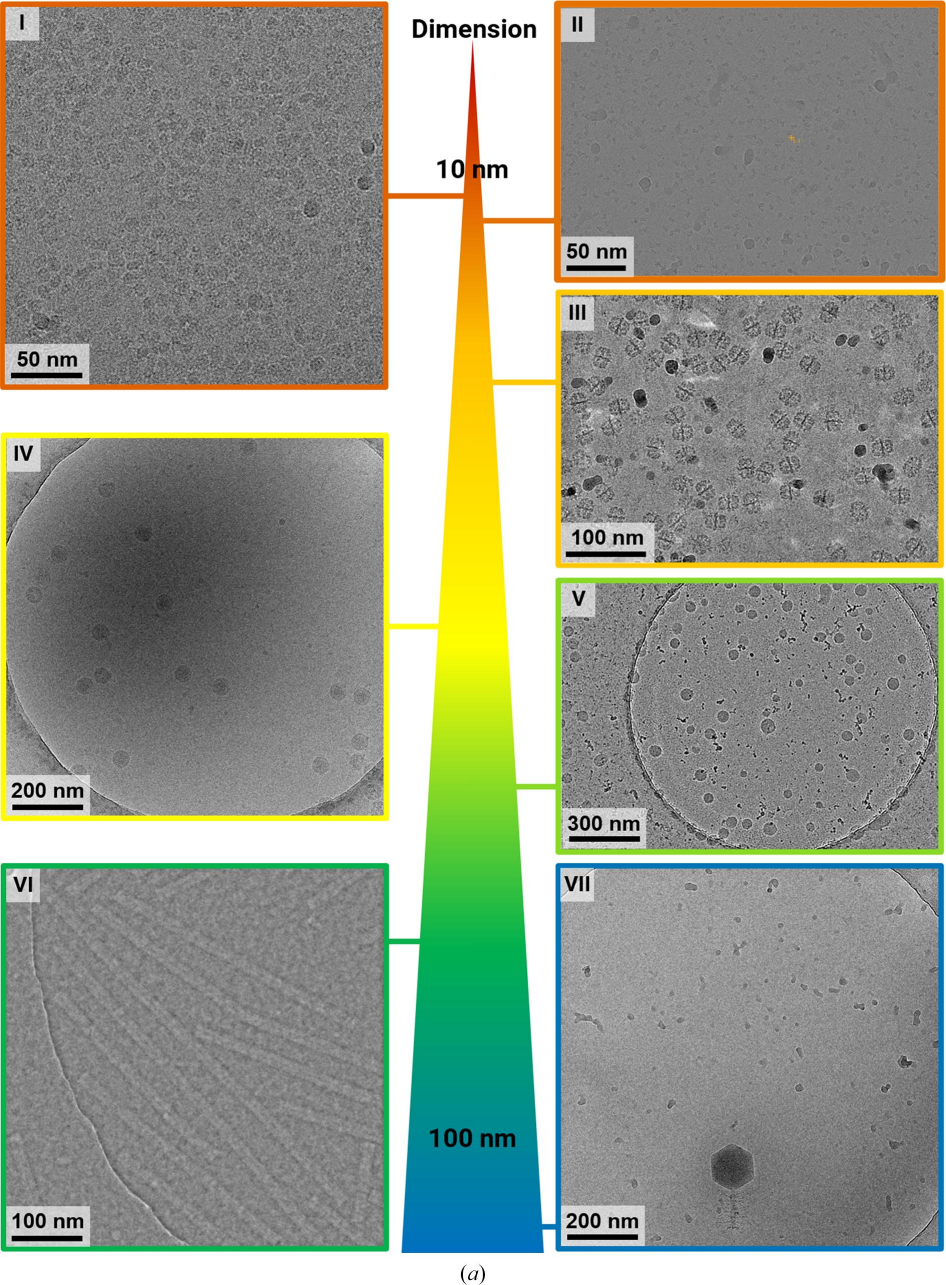

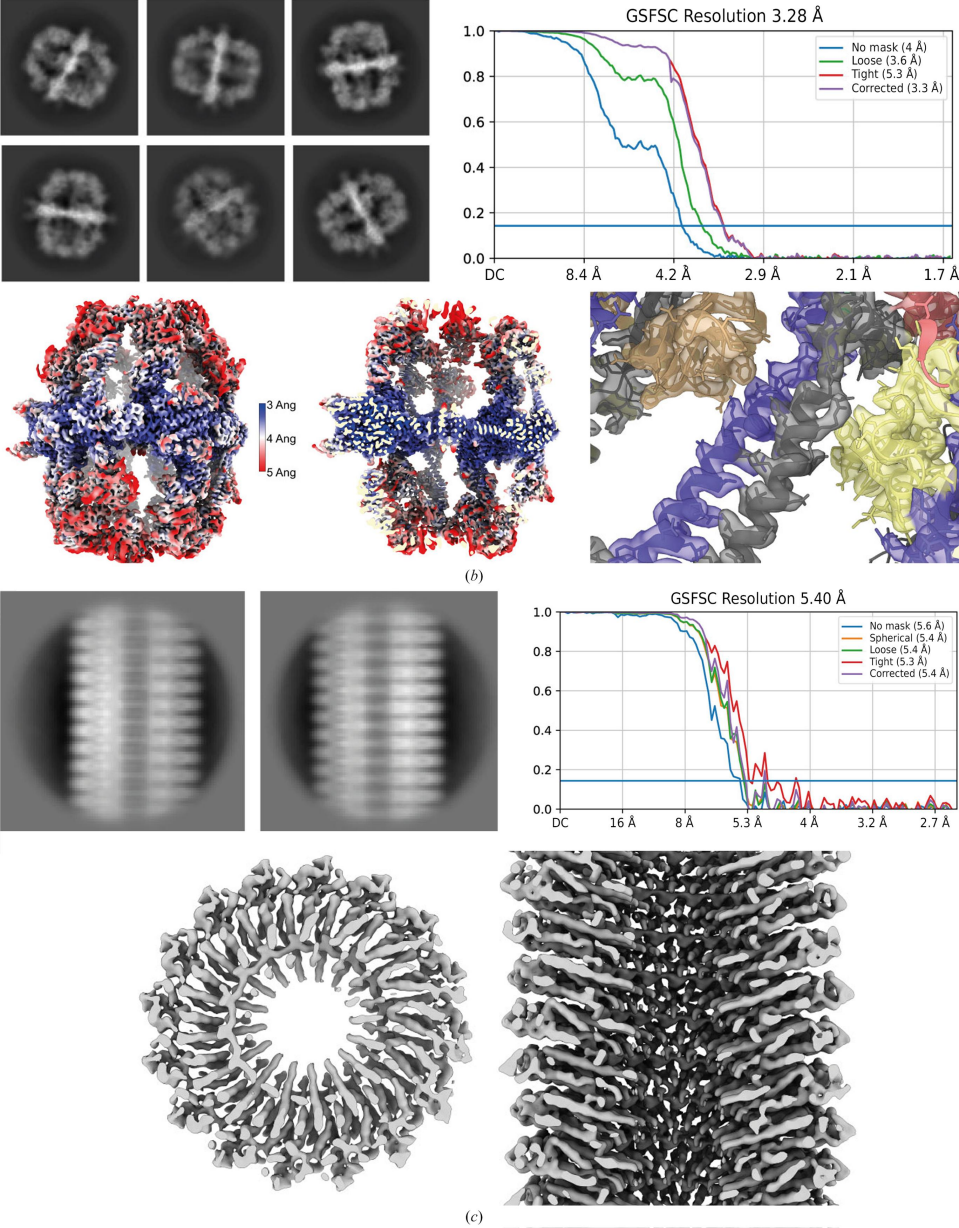
Case studies of the VitroJet with a variety of samples. (*a*) Representative micrographs for use cases with different dimensions. I: membrane proteins with a diameter of 10 nm reconstructed to a resolution of 2.7 Å. II: nucleosome micrograph with a size of 12 nm, which is representative, reconstructed to 3.0 Å resolution. III: fatty-acid synthase (FAS) representative micrograph with a diameter of 27 nm, reconstructed to 3.3 Å resolution. IV: micrograph of tick-borne encephalitis virus with a size of 50 nm. V: representative micrograph of LNPs vitrified with the VitroJet with diameters ranging from 60 to 100 nm. VI: iDPC–STEM micrograph of Tobacco mosaic virus (TMV) samples with vitreous ice micrograph at CSA = 2.0 mrad. VII: micrograph of purified bacteriophage FJy-3 with a capsid diameter of approximately 150 nm. (*b*) Selected 2D classes of FAS exclusively show side views. Fourier shell correlation after nonuniform refinement in *cryoSPARC*, a local resolution map and a slab of local resolution map are shown. The slab of the FAS center with colored reconstructed map and PDB entry 6ta1 shows clear side-chain features. (*c*) Selected 2D classes of the TMV sample reconstructed to an FSC(0.143) resolution of 5.4 Å. The real-space top and side views of the reconstructed map show the clear secondary-structure features that are expected at this resolution.

The purified membrane protein was vitrified on UltraAuFoil grids using the VitroJet. This resulted in a smooth gradient of ice thickness across the pin-printed area. A random particle distribution was observed in micrographs during screening (I in Fig. 4 ▸ *a*). Data collections from two identical grids were combined to produce a respectable 2.7 Å resolution reconstruction with a wide range of particle orientations effectively covered (Supplementary Fig. S1).

The VitroJet streamlines cryo-EM grid preparation significantly and novices can consistently make high-quality grids, due to the parameterized protocol settings and the ability to pre-clip grids at room temperature. While the VitroJet is a sophisticated, high-precision automated sample-preparation machine, further evaluation on different samples is essential to assess its consistency in achieving ice thickness with nanometre accuracy. We speculate that employing pin printing and jet vitrification might yield distinct outcomes compared with traditional methods in specific projects. Furthermore, it is often advantageous to maintain a variety of technologies to cater for the distinct requirements of different projects.

#### 3.3.2. Nucleosome

A nucleosome is a histone–DNA complex with an approximate molecular weight of 250 kDa and a diameter of 12 nm. This sample posed orientation-bias issues on the Vitrobot, decreasing the efficiency of structure determination.

Therefore, grid preparation of this sample was explored on the VitroJet in combination with Quantifoil 200 mesh R2/1 grids. Optimal grids were produced with 30 s of plasma-cleaning, a standoff distance of 10 µm and a velocity of 1 mm s^−1^. These parameters produced grids with approximately ten usable squares for single-particle data collection (II in Fig. 4 ▸ *a*). The data set from a nucleosome-prepared grid resulted in a 3D reconstruction of approximately 3.0 Å resolution (Supplementary Fig. S2). Although orientation bias was still present on the VitroJet, it was apparent to a lesser extent.

#### 3.3.3. Fatty-acid synthase

Yeast fatty-acid synthase (FAS) is an enzyme with a molecular weight of 2.6 MDa and a diameter of 27 nm. It has been shown to adsorb to the air–water interface in unsupported holey films during traditional grid preparation, resulting in protein denaturation of up to 90%. Classification in 2D and 3D showed that density at the distal part was either lacking or only weakly present, limiting particle reconstruction (D’Imprima *et al.*, 2019 ▸). We were interested to determine whether structure determination of this difficult protein is possible with the VitroJet.

Freshly purified FAS was vitrified on UltrAuFoil grids in the VitroJet and the complete deposition area was imaged with a 200 kV Talos Arctica with an energy-filtered K3 camera, generating a total of 3696 micrographs (III in Fig. 4 ▸ *a*). From these images, 45 979 particles were used for a 3D reconstruction with *D*3 symmetry to a resolution of 3.3 Å (Fig. 4 ▸ *b*). The density map shows clear side-chain features with sufficient resolution for atomic model building. 3D classification into three distinct classes with *C*1 symmetry revealed two damaged 3D classes containing 68% of the selected particles and one intact class containing 32% of the selected particles. *D*3 symmetry together with acceptable Fourier space coverage from side views of the intact class still enabled high-resolution structure determination using the VitroJet with grids in the absence of a continuous support film (Supplementary Fig. S3).

#### 3.3.4. Lipid nanoparticles

Lipid nanoparticles (LNPs) represent a highly promising category of drug-delivery systems, as prominently demonstrated in the development and utilization of LNP-based mRNA vaccines to combat COVID-19 during the recent pandemic (Liu *et al.*, 2021 ▸; Hou *et al.*, 2021 ▸; Mitchell *et al.*, 2021 ▸; van der Meel *et al.*, 2021 ▸; Schoenmaker *et al.*, 2021 ▸; Baden *et al.*, 2021 ▸). LNPs offer a dependable and customizable mechanism for drug delivery, ensuring not only the precise delivery of RNA to its intended target, but also safeguarding the nucleic acid (Álvarez-Benedicto *et al.*, 2022 ▸). In the context of their practical application, the size and encapsulation efficiency of LNPs are pivotal considerations during design for *in vivo* use, into which cryo-EM can provide valuable insights (Kulkarni *et al.*, 2018 ▸). In this case, we focused on ionizable LNPs designed to encapsulate siRNA, which aim to disrupt the expression of NEMO/IKK-γ mRNA. The diameter of these LNPs is approximately 60–100 nm. In our initial efforts to vitrify LNPs, we observed a strong attraction of LNPs to the carbon support of the grid. These LNPs selectively adhered to the edges of the foil holes, presumably because of the thicker ice gradient in this region. Achieving a consistently thicker ice layer on continuous carbon support grids using blotting presented challenges.

In this instance, we prepared LNP samples on Quantifoil grids with a continuous 2 nm carbon support film. The grids were processed in the VitroJet using 30 s of plasma-cleaning and were deposited at speeds ranging from 2 to 5 mm s^−1^. Cryo-EM imaging demonstrated the size distribution of LNPs (V in Fig. 4 ▸ *a*) and unveiled further insights into the morphology of the samples. The gentle built-in plasma technology and controlled protocols of the VitroJet yielded a reproducible ice thickness suitable for LNPs (Supplementary Fig. S4). This method, in combination with the graphical user interface, which is quite suitable for novice users, allows a robust workflow for LNP-based projects to be established.

#### 3.3.5. Tobacco mosaic virus

Due to its high order and internal symmetry, RNA-loaded Tobacco mosaic virus (TMV) is an ideal test sample for cryo-EM methods development (Ruska *et al.*, 1939 ▸; Fromm *et al.*, 2015 ▸; Kruger *et al.*, 2000 ▸). TMV forms helical rods with a diameter of 18 nm and variable lengths. While the molecular weight of the symmetry unit is only 18.6 kDa, the rod assemblies with a helical rise of 1.4 Å can reach dozens of MDa. We recently used TMV for near-atomic resolution determination of cryo-specimens from scanning transmission electron microscopy using integrated phase contrast (iDPC–STEM; Lazić *et al.*, 2016 ▸, 2022 ▸; Lazić & Bosch, 2017 ▸; Yücelen *et al.*, 2018 ▸). The increased viscosity of the sample solution occasionally results in nonvitreous ice when plunge-freezing, which is observed in STEM images as bright spots originating from Bragg reflections.

The sample was prepared on Quantifoil R2/1 grids with the VitroJet, and a data set of 103 iDPC–STEM micrographs at a convergence semi-angle (CSA) of 2.0 mrad was collected (VI in Fig. 4 ▸ *a*). From this data set, 13 644 particles were selected to obtain a 3D reconstruction with a resolution of 5.4 Å with imposed helical symmetry (Fig. 4 ▸ *c*). In STEM, the maximum obtainable resolution depends on the probe diameter and the associated incident CSA (Lazić *et al.*, 2022 ▸; Bosch & Lazić, 2019 ▸). The obtained resolution of 5.4 Å at CSA = 2.0 mrad is now at the maximum theoretical STEM resolution [λ/2 × CSA, λ(300 kV) = 1.969 pm] for this CSA. This result improves our previous reconstruction resolution at this CSA of FSC(0.143) = 6.3 Å from 20 micrographs and 2073 particles (Lazić *et al.*, 2022 ▸). The reconstructed map resolves the expected secondary-structure features. Jet vitrification reduced areas with nonvitreous ice and improved the sample for this data collection (Supplementary Fig. S5).

#### 3.3.6. Viruses

We tested the VitroJet in combination with a wide range of particle sizes, including purified proteins or protein complexes ranging from 54 kDa to several megadaltons on Quantifoil grids. Grid preparation using the VitroJet with small proteins and macromolecular complexes such as ribosomes worked satisfactorily with the original 120 µm diameter pins; however, the deposition of large viral particles was challenging.

After increasing the pin diameter to 150 µm, a greater sample volume is picked up and deposited. This enlarges the area covered by pin printing and increases the probability of depositing large macromolecules (Supplementary Fig. S6). In the case of the 50 nm tick-borne encephalitis virus used in this study, we were able to detect up to 18 particles per hole (IV in Fig. 4 ▸ *a*). For giant viruses, such as the bacteriophage FJy-3 with a capsid diameter of approximately 150 nm and a 120 nm tail, one particle per hole could be detected in 16 of 32 holes (VII in Fig. 4 ▸ *a*). Due to the larger pin diameter, the deposition and vitrification of relatively large spherical viral particles also worked efficiently.

## 4. Discussion

The presented use cases demonstrate a range of sample types for which the VitroJet can enable and streamline the sample-preparation and screening process. However, they have also highlighted the unique requirements of each sample type and the potential need for optimization. Depending on the sample requirements, specific grid types and preparation techniques can result in distinct outcomes. In practice, it often remains unclear why a specific sample yields better results when using different preparation methods. Nevertheless, process reproducibility is expected to be valuable in optimizing the combination of biochemistry, sample carrier and grid preparation in a structured manner.

Several distinct grid-preparation devices have been commercialized and made available to the field. The Chameleon, based on Spotiton, pursues minimization of the time between deposition and vitrification by using inkjet deposition onto special nanowire grids (Dandey *et al.*, 2018 ▸). Although it has been demonstrated to help in reducing preferred orientations in some cases, in others it had no or the opposite effect, suggesting that deleterious interactions with the air–water interface cannot always be outrun using the current technology (Klebl *et al.*, 2020 ▸; Noble *et al.*, 2018 ▸). The CryoWriter, which uses capillary-based writing and evaporation, enables microfluidic isolation or manipulation prior to deposition, presenting alternative approaches for handling sensitive samples (Schmidli *et al.*, 2019 ▸). The CryoGenium is applicable both to single-particle analysis as well as correlative light and electron microscopy, and is based on dipping a grid into the sample solution followed by sample removal through capillary suction (Koning *et al.*, 2022 ▸). Along with the VitroJet, all four methods contain an integrated glow discharger to reduce manual handling and include a camera for quality monitoring. Nevertheless, the ability to process and vitrify Autogrids in the VitroJet eliminates the need for clipping Autogrids under cryogenic conditions after vitrification. Additionally, the VitroJet camera permits quantitative ice-thickness estimation in the holes, which plays an important role in high-resolution cryo-EM data collection (Kim *et al.*, 2018 ▸; Neselu *et al.*, 2023 ▸; Henderikx *et al.*, 2023 ▸).

With the maturation of single-particle analysis as a mainstream technique for structure determination, it is expected that throughput, reproducibility and ease of use will become increasingly important. In the VitroJet grids can be processed fully automatically, although the user still needs to confirm the protocol parameters for each grid. Being able to predefine settings for all 12 Autogrids would allow complete unattended vitrification experiments, where the camera images can be used to generate thickness maps for quality assessment. These results can be used to prioritize screening in the microscope. Enabling transfer of the thickness maps measured by the VitroJet camera will reduce atlas acquisition and screening time in the electron microscope. With the known relationship between protocol settings and thickness, one could develop algorithms to autonomously optimize deposition parameters on the fly without user interaction.

The increasing standardization in cryo-EM drives the progression of research to other fascinating topics, such as time-resolved cryo-EM and cryo-electron tomography (cryo-ET). Typical plunge-freezing devices insert grids into liquid ethane at a velocity of 1 m s^−1^ for vitrification, meaning that the immersion of a 3 mm EM grid takes 3 ms, similar to the timescales of many protein reactions (Mäeots & Enchev, 2022 ▸; Klebl *et al.*, 2023 ▸; Engstrom *et al.*, 2021 ▸). In jet vitrification, the ethane jet is targeted onto the grid at a specific location and time, which could enable the cryofixation of samples with higher spatial and time resolution. In cell vitrification for cryo-ET, plunge-freezing often results in crystalline ice due to the larger sample thickness. Higher cooling rates from jet vitrification might yield an advantage in cell vitrification for cryo-ET, enabling the vitrification of larger samples (Berger, Ravelli, López-Iglesias, Kudryashev *et al.*, 2021 ▸; Berger, Ravelli, López-Iglesias & Peters, 2021 ▸).

The VitroJet prototype provided a proof of concept showing advantages in both minimizing operator dependency and improving reproducibility over the grid-preparation process. All lessons learned were implemented in the instrument that is presented here, such as the plasma-module materials, temperature-controlled climate chamber, optical ice-thickness estimation and automated filling of cryogens. The described technical advances aim to ameliorate the grid-preparation process and share the benefits of the workflow with other scientists. Together, the results demonstrate the evolution of the VitroJet with increased control and automation, allowing novice operators to produce consistent samples with good quality.

## Supplementary Material

EMDB reference: fatty-acid synthase, EMD-19477

EMDB reference: Tobacco mosaic virus, EMD-19489

Supplementary Figures. DOI: 10.1107/S2059798324001852/ih5005sup1.pdf

Supplementary Movie S1: sample pickup. DOI: 10.1107/S2059798324001852/ih5005sup2.avi

Supplementary Movie S2: deposition. DOI: 10.1107/S2059798324001852/ih5005sup3.avi
