# Supplementary material for: VitroJet: new features and case studies
Source: Acta Crystallogr D Struct Biol. 2024 Mar 15;80(Pt 4):232–46. doi: 10.1107/S2059798324001852 (PMC10994172; doi:10.1107/S2059798324001852)
Supplement: Supplementary file 1 [file d-80-00232-sup1.pdf]

STRUCTURAL BIOLOGY

**Volume 80 (2024)**

**Supporting information for article:**

## VitroJet: new features and case studies

**Rene J. M. Henderikx, Daniel Mann, Aušra Domanska, Jing Dong, Saba Shahzad, Behnam Lak, Aikaterini Filopoulou, Damian Ludig, Martin Grininger, Jeffrey Momoh, Elina Laanto, Hanna M. Oksanen, Kyrylo Bisikalo, Pamela A. Williams, Sarah J. Butcher, Peter J. Peters and Bart W. A. M. M. Beulen**

## Membrane protein

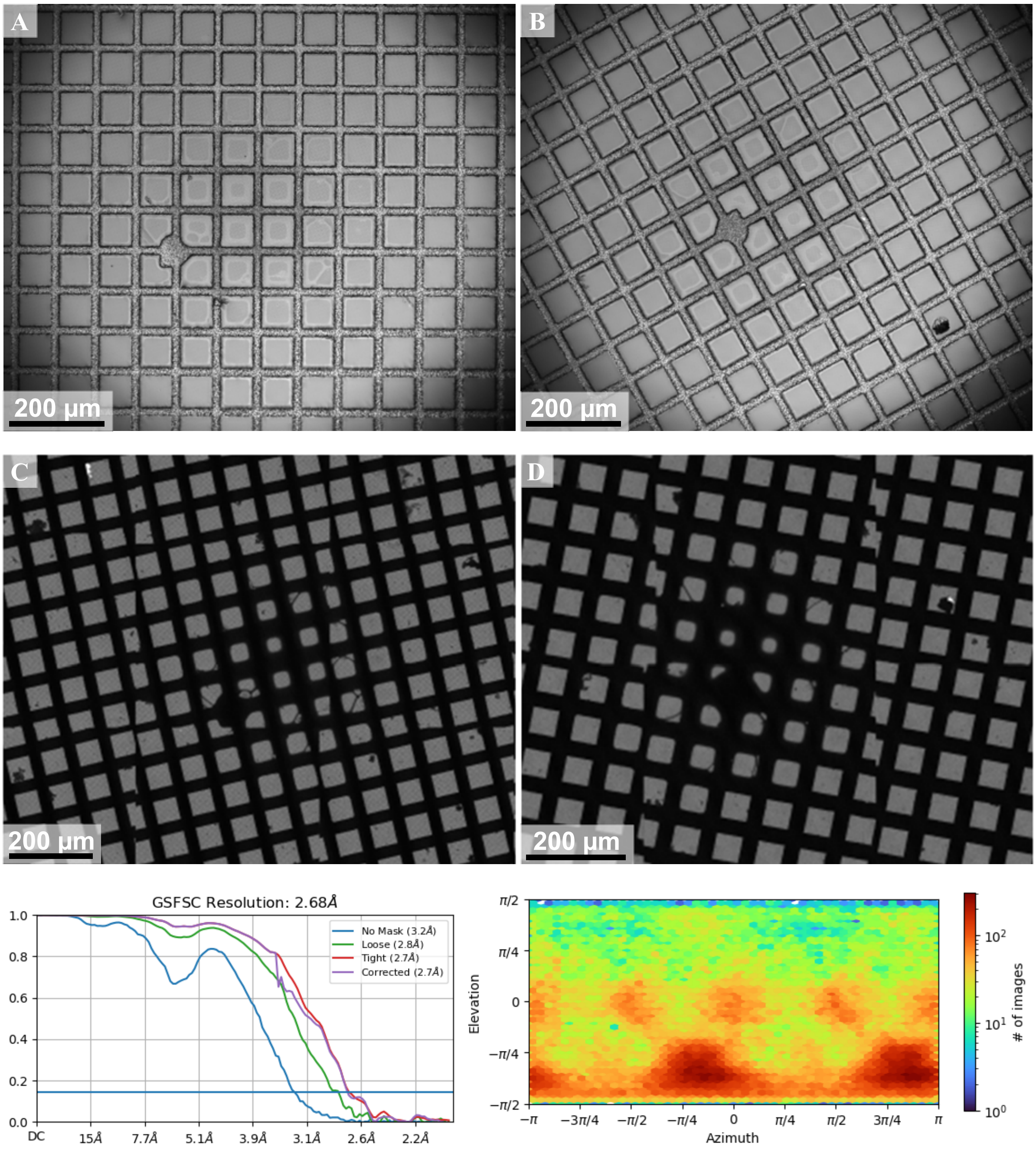


**Figure S1** Additional information on the VitroJet case study with a membrane protein. (AB) Images from the VitroJet grid camera after deposition of the membrane protein on UltraAuFoil grids. (CD) Low magnification cryo-EM atlases from the grids for data collection and 3D reconstruction. (E) Graph with the FSC(0.143) curve demonstrating the achieved resolution. (F) Orientational distribution of the particles in the 3D reconstruction.

**Nucleosome**

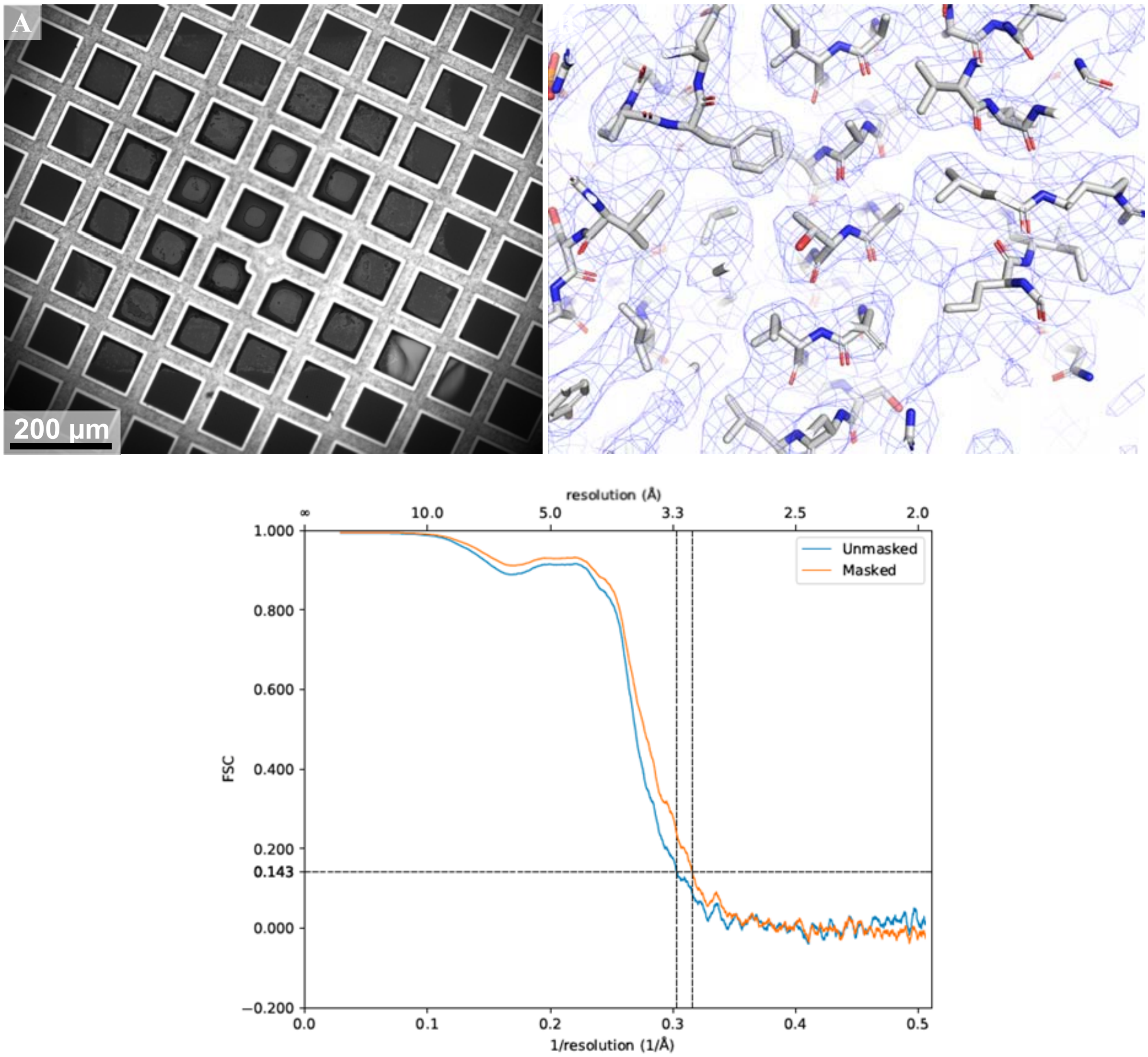


**Figure S2** Additional information on the VitroJet case study with a nucleosome sample. (A) Image from the VitroJet grid camera after deposition of the nucleosome on a Quantifoil grid. (B) Segment of the density map that was reconstructed showing the detail of the side chains that are resolved. (C) Graph with the FSC(0.143) curve demonstrating the achieved resolution.

**Fatty acid synthase**

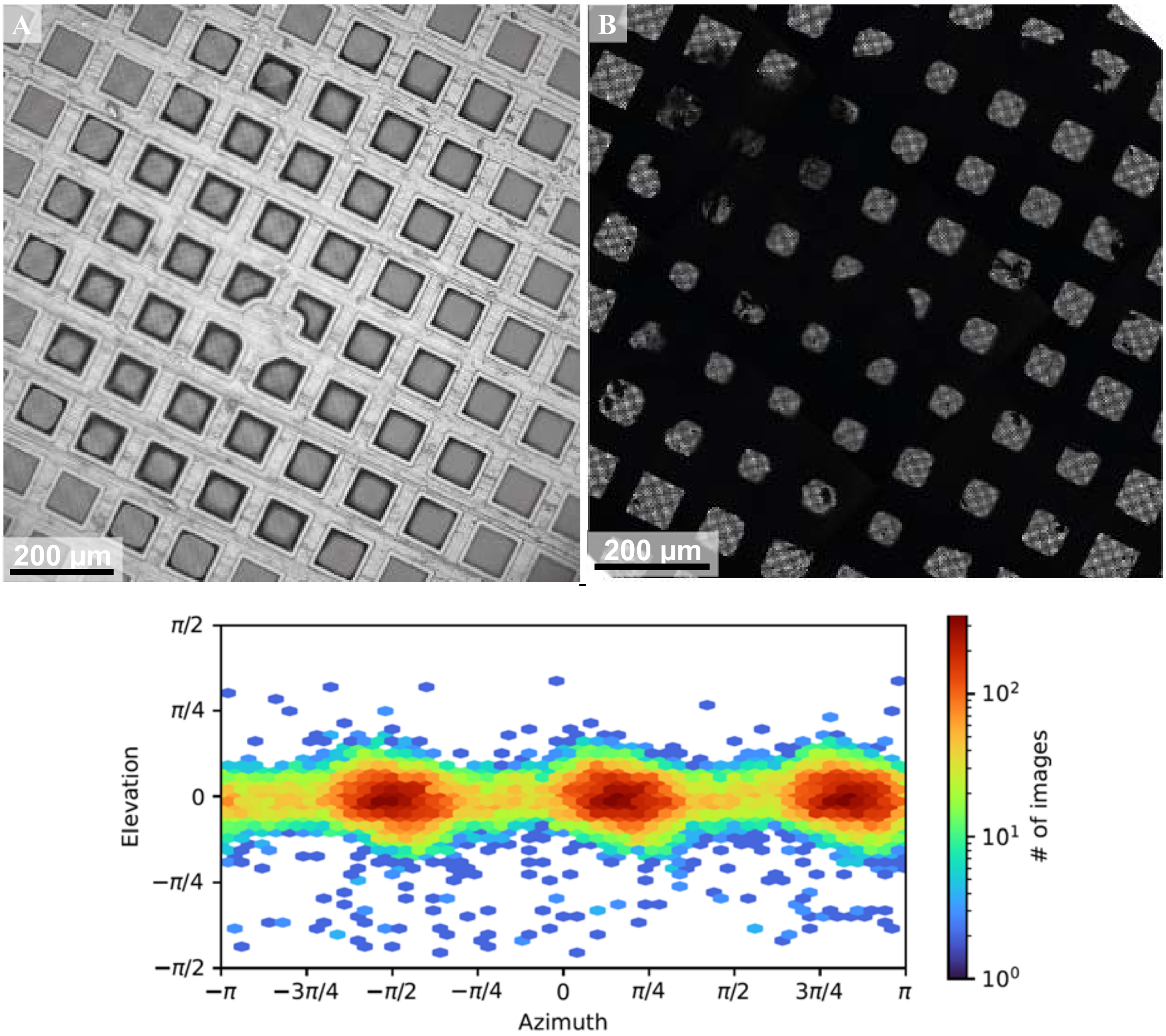


**Figure S3** Additional information on the VitroJet case study with *fatty acid synthase*. (A) Image from the VitroJet grid camera after deposition of fatty acid synthase on a UltraAuFoil grid. (B) Low magnification cryo-EM atlas of the grid for data collection and 3D reconstruction. (C) Orientational distribution of the particles in the 3D reconstruction.

**Lipid nanoparticles**

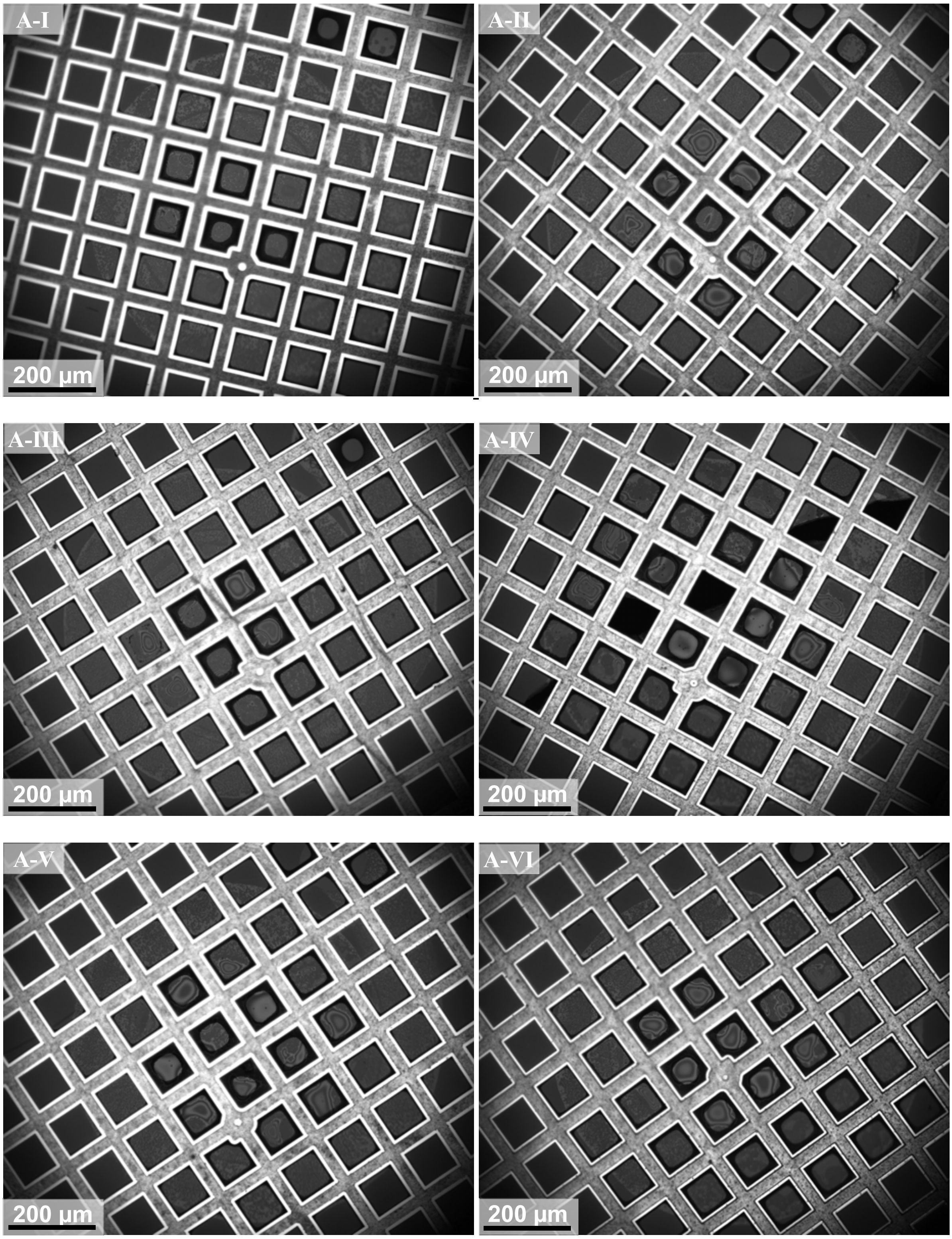

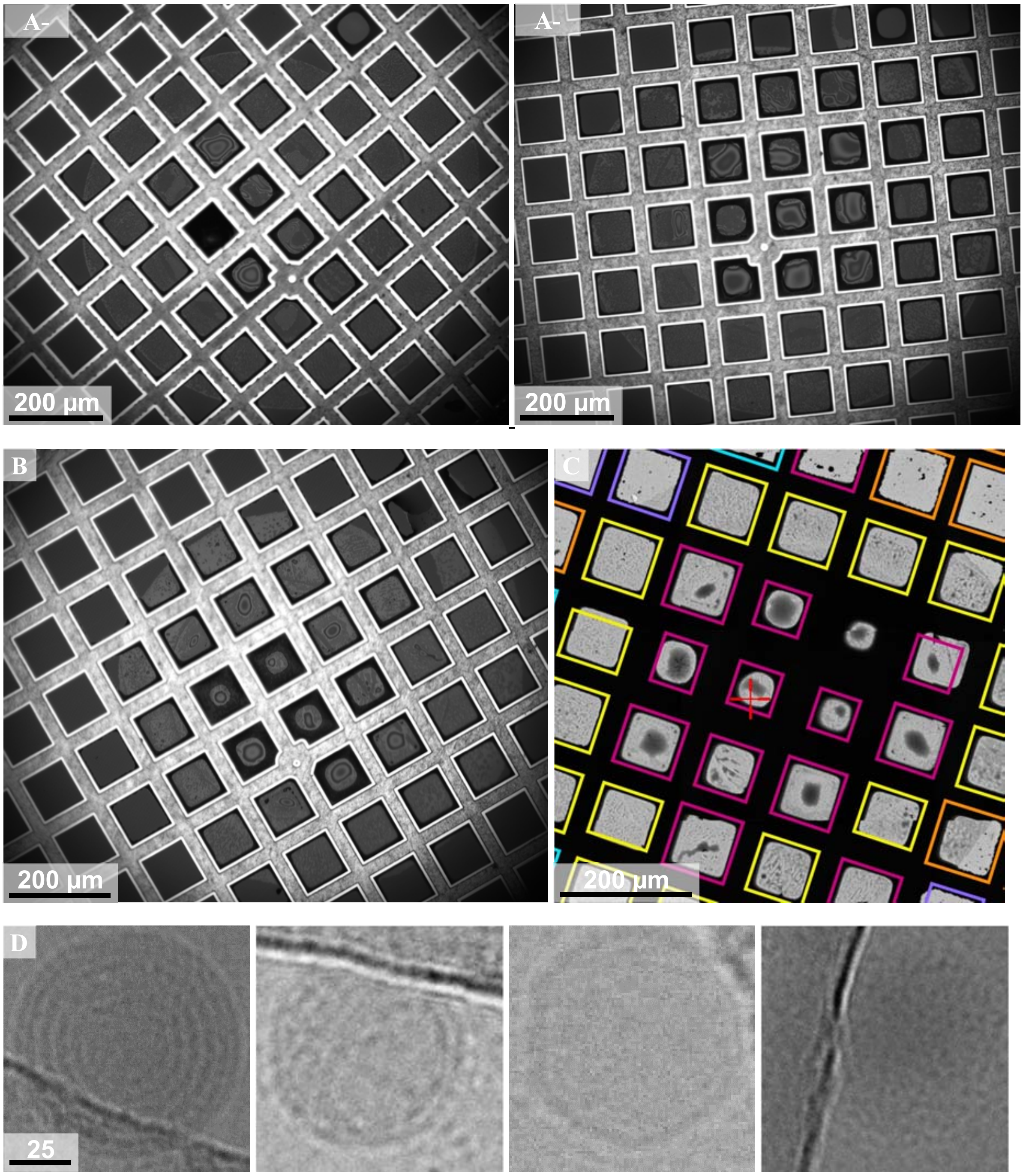


**Figure S4** Additional information on the VitroJet case study with lipid nanoparticles. (A) Images from the VitroJet grid camera after deposition lipid nanoparticles on Quantifoil grids demonstrating reproducibility. (B) VitroJet grid camera image that is used for further analysis. (C) Low magnification cryo-EM atlas of the grid for further analysis. (D) High resolution cryo-EM images of different LNP samples, where a difference in morphology and internal lamellarity can be observed.

## Tobacco mosaic virus

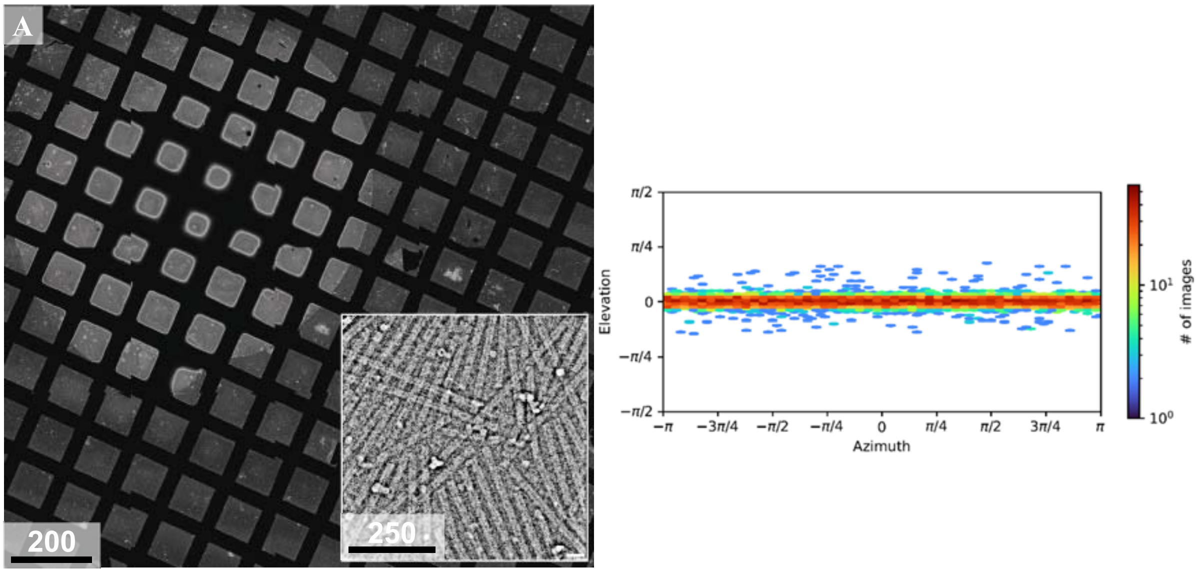


**Figure S5** Additional information on the VitroJet case study with tobacco mosaic virus. (A) Low magnification cryo-EM atlas in HAADF-STEM of an UltraAuFoil grid used for data collection and reconstruction. The pin printed spiral is visible in white against the grey carbon background. Inset: iDPC-STEM micrograph at CSA=2.0 mrad. (B) Orientational distribution of the particles in the 3D reconstruction. Due to the aspect ratio between length and diameter of the fibres, the orientational distribution of the helical reconstruction is flat.

## Viruses

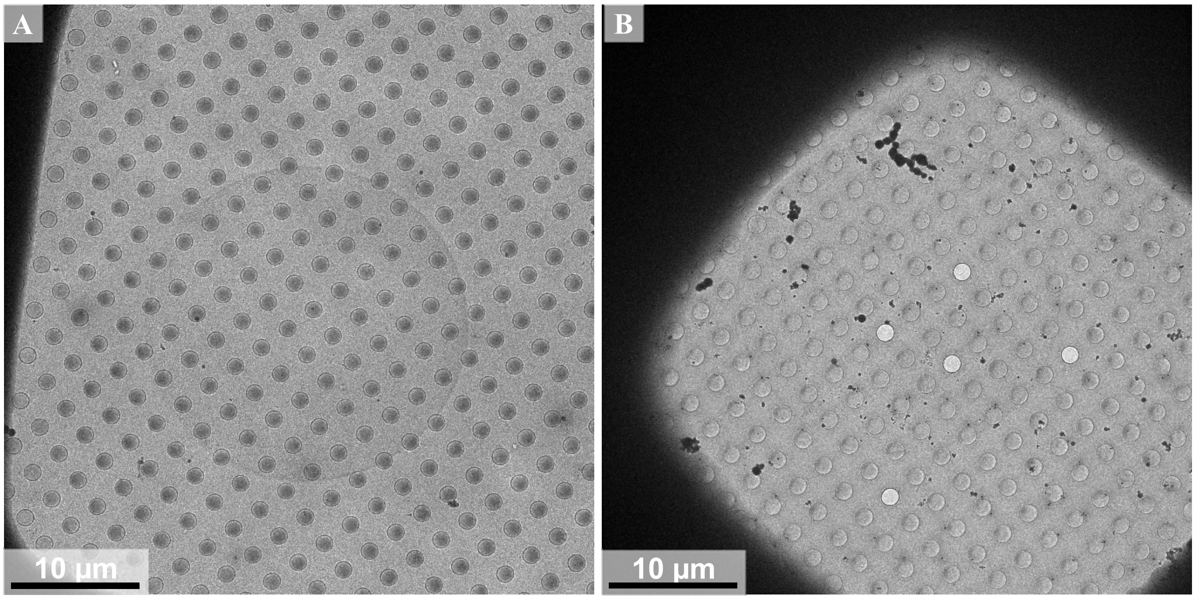


**Figure S6** Additional information on the VitroJet case study with large viruses. (A) Medium magnification cryo-EM square image of the Quantifoil grid prepared with the tick-borne encephalitis virus on the VitroJet. (B) Medium magnification cryo-EM square image of the Quantifoil grid prepared with the bacteriophage FJy-3 on the VitroJet.
